# Correlation of blood biomarkers with microstructural brain injury in survivors of out-of-hospital cardiac arrest

**DOI:** 10.1093/braincomms/fcag378

**Published:** 2026-10-08

**Authors:** Mikael V Laaksonen, Jussi P Posti, Carita C Hollmén, Kaj Blennow, Henrik Zetterberg, Jaakko K Rinne, Sami M Virtanen, Jani P Saunavaara, Riitta K Parkkola, Ruut M Laitio, Olli J Arola, Marja S Hynninen, Emmi K Ylikoski, Juha A Martola, Marjaana S Tiainen, Risto O Roine, Harry Scheinin, Mervyn Maze, Timo Roine, Tero Vahlberg, Timo T Laitio, Xe-HYPOTHECA Working Group, Juhani Airaksinen, Juha Grönlund, Veli-Pekka Harjola, Outi Inkinen, Kirsi Korpi, Jussi Niiranen, Eija Nukarinen, Klaus T Olkkola, Mikko Pietilä, Antti Saraste, Päivi Silvasti, Marjut Varpula, Johanna Wennervirta

**Affiliations:** Department of Perioperative Services, Intensive Care Medicine and Pain Management, Turku University Hospital and University of Turku, 20521 Turku, Finland; Neurocenter, Department of Neurosurgery and Turku Brain Injury Center, Turku University Hospital and University of Turku, 20521 Turku, Finland; Department of Radiology, University of Turku, Turku University Hospital, 20521 Turku, Finland; Department of Psychiatry and Neurochemistry, Institute of Neuroscience and Physiology, The Sahlgrenska Academy at the University of Gothenburg, S-431 80 Mölndal, Sweden; Clinical Neurochemistry Laboratory, Sahlgrenska University Hospital, S-431 80 Mölndal, Sweden; Paris Brain Institute, ICM, Pitié-Salpêtrière Hospital, Sorbonne University, 75646 Paris Cedex 13, France; Neurodegenerative Disorder Research Center, Division of Life Sciences and Medicine, Department of Neurology, Institute on Aging and Brain Disorders, University of Science and Technology of China and First Affiliated Hospital of USTC, Hefei 230026, China; Department of Psychiatry and Neurochemistry, Institute of Neuroscience and Physiology, The Sahlgrenska Academy at the University of Gothenburg, S-431 80 Mölndal, Sweden; Clinical Neurochemistry Laboratory, Sahlgrenska University Hospital, S-431 80 Mölndal, Sweden; Department of Neurodegenerative Disease, UCL Institute of Neurology, London WC1E 6BT, UK; UK Dementia Research Institute at UCL, London WC1E 6BT, UK; Hong Kong Center for Neurodegenerative Diseases, The Hong Kong University of Science and Technology, Hong Kong, China; Wisconsin Alzheimer’s Disease Research Center, University of Wisconsin School of Medicine and Public Health, University of Wisconsin-Madison, Madison, WI 53792-2420, USA; Neurocenter, Department of Neurosurgery and Turku Brain Injury Center, Turku University Hospital and University of Turku, 20521 Turku, Finland; Department of Radiology, University of Turku, Turku University Hospital, 20521 Turku, Finland; Department of Medical Physics, Turku University Hospital and University of Turku, 20521 Turku, Finland; Department of Radiology, University of Turku, Turku University Hospital, 20521 Turku, Finland; Department of Perioperative Services, Intensive Care Medicine and Pain Management, Turku University Hospital and University of Turku, 20521 Turku, Finland; Department of Perioperative Services, Intensive Care Medicine and Pain Management, Turku University Hospital and University of Turku, 20521 Turku, Finland; Division of Intensive Care Medicine, Department of Anesthesiology, Intensive Care and Pain Medicine, University of Helsinki and Helsinki University Hospital, 00029 Helsinki, Finland; Division of Intensive Care Medicine, Department of Anesthesiology, Intensive Care and Pain Medicine, University of Helsinki and Helsinki University Hospital, 00029 Helsinki, Finland; Department of Radiology, University of Helsinki and Helsinki University Hospital, 00029 Helsinki, Finland; Department of Neurology, University of Helsinki and Helsinki University Hospital, 00029 Helsinki, Finland; Division of Clinical Neurosciences, University of Turku, Turku University Hospital, 20521 Turku, Finland; Department of Perioperative Services, Intensive Care Medicine and Pain Management, Turku University Hospital and University of Turku, 20521 Turku, Finland; Department of Anesthesia and Perioperative Care, University of California, San Francisco, CA 94143, USA; Department of Neuroscience and Biomedical Engineering, Aalto University School of Science, 00076 Espoo, Finland; Turku Brain and Mind Center, University of Turku, 20014 Turku, Finland; Department of Biostatistics, University of Turku, 20014 Turku, Finland; Department of Perioperative Services, Intensive Care Medicine and Pain Management, Turku University Hospital and University of Turku, 20521 Turku, Finland

**Keywords:** out-of-hospital cardiac arrest, xenon, diffusion tensor, neurofilament light chain, white matter

## Abstract

In-hospital mortality among resuscitated out-of-hospital cardiac arrest (OHCA) patients remains high. Identifying patients who will benefit from intensive care while avoiding prolonged, potentially unnecessary treatments is essential. Blood-based biomarkers provide a novel method for evaluating brain injury. The ability of glial fibrillary acidic protein (GFAP), neurofilament light chain (NfL), neuron-specific enolase (NSE) and total-tau (t-tau) to measure white and grey matter injury is unestablished in comatose OHCA survivors treated with targeted temperature management (TTM) or xenon combined with TTM. We examined correlations between the biomarkers and white and grey matter injury assessed with diffusion tensor imaging (DTI). This was a randomized, single-blind, phase 2 clinical drug trial involving two multipurpose intensive care units in Finland. It was conducted between August 2009 and March 2015. One hundred and ten comatose OHCA patients aged 24 to 76 years were randomly assigned to either inhaled xenon treatment combined with 24-h TTM (33°C) (*n* = 55, xenon group) or TTM alone (*n* = 55, control group). MRI data were available from 96 patients, with a median imaging time of 53 h (interquartile range, 47–64 h) after OHCA. Biomarker data were obtained from 105 patients. In the control group of 48 patients, after adjusting for age, sex and site, a disruption of white matter microintegrity as measured by decreased fractional anisotropy (FA) of DTI, the primary endpoint of the trial, showed a significant negative correlation with NfL at 24, 48 and 72 h (ρ = −0.28 to −0.37; *P* < 0.05), NSE at 48 and 72 h (ρ = −0.30 to −0.35; *P* < 0.05) and t-tau only at 72 h (ρ = −0.30; *P* < 0.05). In the xenon group (*n* = 48), these correlations were absent at all timepoints. The NfL–FA partial correlations showed progressive divergence between the xenon and control groups, with a statistically significant between-group difference observed at 72 h (ρ = −0.37 versus ρ = 0.14; *P* = 0.016). Cortical or basal ganglia grey matter mean diffusivity did not have a consistent correlation with biomarker levels at different time points. No statistically significant differences were found between the xenon and control groups in the adjusted geometric mean levels of GFAP, NfL, NSE or t-tau at various time points following OHCA. In conclusion, NfL correlated with FA in controls but not in the xenon group. This between-group difference reached significance at 72 h, suggesting that xenon may modify the relationship between axonal injury and white matter microstructure after OHCA.

## Teaser

Laaksonen *et al*. report that circulating brain injury markers correlated with white matter injury on MRI in resuscitated out-of-hospital cardiac arrest patients receiving standard care, but not in those treated with xenon. The authors interpret this finding as possibly reflecting a drug effect.

## Graphical Abstract

**Graphical Abstract:**
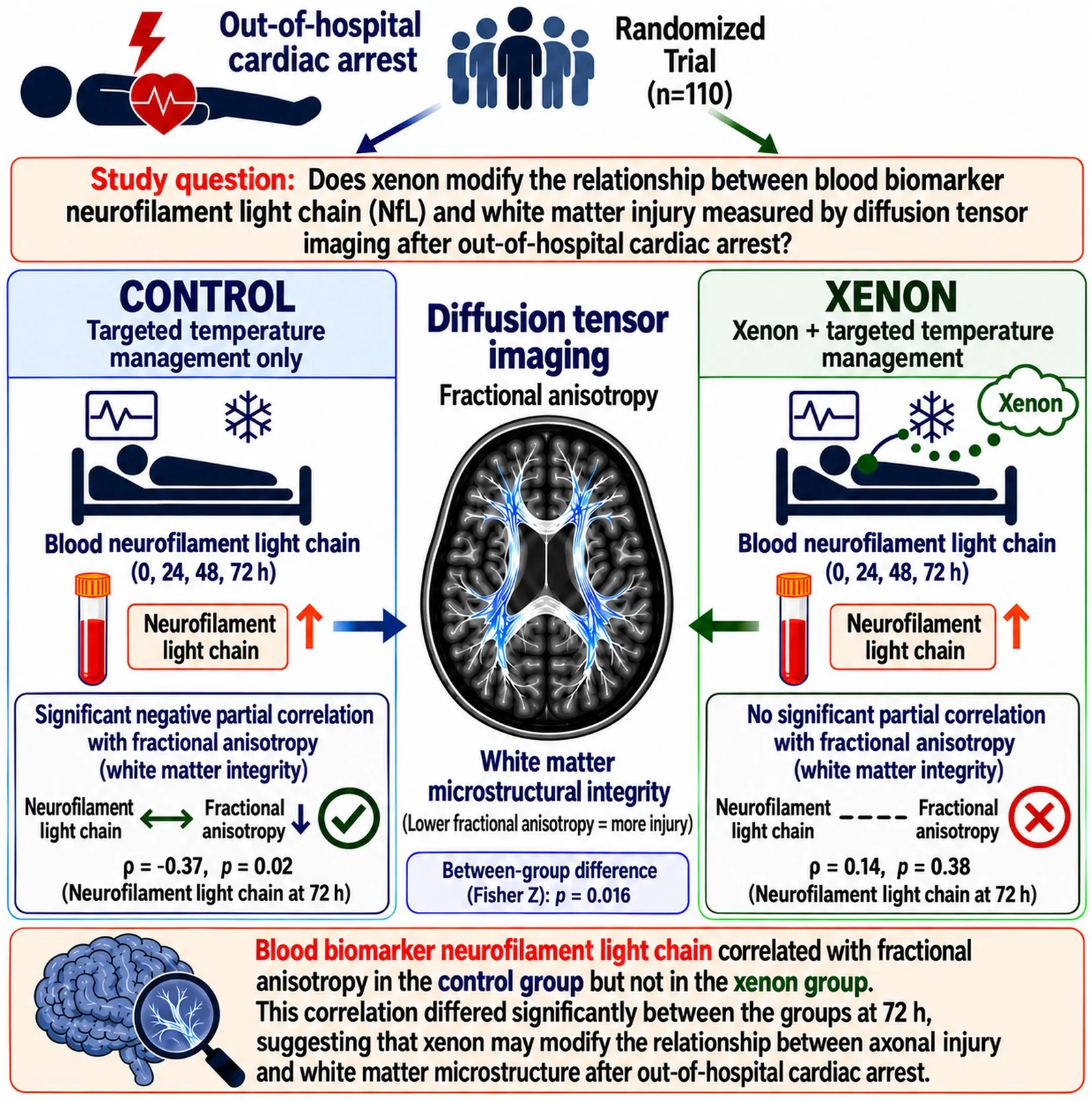

## Introduction

The in-hospital mortality rate for successfully resuscitated out-of-hospital cardiac arrest (OHCA) patients remains high, fluctuating between 41% and 86%.^1,2^ Post-cardiac arrest brain injury is the most common cause of mortality and the leading cause of long-term morbidity in resuscitated patients.^1^ Therefore, it is crucial to identify those who benefit from intensive care and, at the same time, to limit prolonged suffering with aggressive treatments.

OHCA results in widespread ischaemia and subsequent global brain damage.^3^ The microstructural consequences of this injury can be assessed using diffusion tensor imaging (DTI), an MRI technique that enables *in vivo* quantification of white and grey matter integrity by characterizing the directionality and magnitude of water diffusion within a voxel.^4,5^ In white matter, diffusion is constrained by axons and cellular membranes,^6^ yielding scalar values such as mean diffusivity (MD), axial diffusivity (AD), radial diffusivity (RD) and fractional anisotropy (FA), the latter reflecting the degree of directional coherence of water motion (values between 0 and 1). The value 0 indicates fully random water diffusion, while a value of 1 represents a fully unidirectional diffusion.^7^ A high FA value indicates highly directional water motion, as seen in healthy and fully matured white matter of an adult human brain.^8^ While DTI has been widely applied in conditions including traumatic brain injury, stroke and ischaemic brain injury, the relationship between serum biomarkers and DTI parameters following OHCA remains poorly understood, particularly during the early phase (≤72 h after the insult). Moreover, blood-based biomarkers for acute diagnosis and outcome prediction are scarce in OHCA patients and even more limited in those treated with xenon, in contrast to traumatic brain injury, where biomarker research has been ongoing for more than two decades.^9^

Unlike OHCA, TBI typically begins with localized brain damage that may later evolve into a more diffuse injury, resulting in broader effects.^10,11^ Hossain *et al*.^12^ investigated the relationship between early biomarker levels and white matter damage as assessed by DTI, with imaging conducted at a median of 8 months post-mild TBI. Similarly, Newcombe *et al*.^13^ examined the association between white matter damage and biomarkers in TBI patients, performing imaging at 8 months and 5 years post-injury. Both studies demonstrated a significant negative correlation between NfL levels and DTI parameters.

Xenon, an *N*-methyl-D-aspartate (NMDA) receptor antagonist, acts as a competitive inhibitor at the glycine site associated with the receptor. NMDA receptors, expressed in white matter myelin, are activated during ischaemia.^14,15^ Once identified as a non-competitive antagonist of the NMDA subtype of glutamate receptors,^16^ xenon was shown in several preclinical studies across three different animal species and various models of acute neuronal injury to have neuroprotective effects. These neuroprotective effects are most pronounced when xenon is administered shortly after injury, within a window of 2–6 h.^16-23^

In 2016, findings of the Xe-HYPOTHECA trial (as listed on Clinicaltrials.gov NCT00879892) showed that xenon could mitigate white matter damage following global ischaemia after a cardiac arrest.^24^ The primary reason for this effect was a reduced degree of white matter injury with those treated with xenon compared to the control group. Evidence for this came from the noticeably elevated global FA in the DTI of white matter pathways in patients administered with xenon compared to those in the control group. However, we have yet to examine the ability of commonly studied blood-based brain injury biomarkers to reflect microstructural white and grey matter damage, as well as the effect of xenon on these biomarker levels in OHCA patients.

Our study aimed to investigate the global ischaemic impact during the early phase (up to 72 h post-OHCA) by evaluating the ability of glial fibrillary acidic protein (GFAP), neurofilament light chain (NfL), neuron-specific enolase (NSE) and total-tau (t-tau) to measure white and grey matter injury through their correlations with DTI parameters. Additionally, we aimed to explore differences in biomarker levels between the control group and the xenon-treated group.

## Materials and methods

### Study design

This study is part of a randomized two-group single-blind phase 2 clinical trial in two academic intensive care units in Finland. The study was approved by the Ethics Committee of the Hospital District of Southwest Finland and by the institutional review boards of Helsinki University Hospital and the Finnish Medicines Agency. An independent data and safety monitoring committee reviewed the data after each enrolment of four patients and after each 6-month interval. The study was conducted in accordance with good clinical practice and the current version of the Declaration of Helsinki.^25^

Patients were recruited from Turku University Hospital, Turku, Finland, between August 2009 and September 2014, and from Helsinki University Hospital, Helsinki, Finland, between October 2012 and September 2014. The 6-month follow-up was completed in March 2015.

Written informed consent was obtained from the patient’s next of kin or legal representative within 4 h of hospital admission. The next of kin were informed of the right to withdraw from the study at any time, but that data collected up to the time of possible withdrawal could be used for analyses specified in the study protocol. Patients were informed accordingly when they regained consciousness.

Patients were assigned in a 1:1 ratio with random block sizes of 4, 6 and 8 to either therapeutic hypothermia treatment alone for 24 h (defined as the control group) or inhaled xenon (LENOXe, Air Liquide Medical GmbH) in combination with hypothermia for 24 h (defined as the xenon group). Allocation to treatment was randomized by computer. Sealed envelopes numbered separately were used in the two centres for randomization, which occurred after informed consent was obtained.

As defined in the amended protocol, secondary endpoints included the intent to explore correlation between the biomarkers GFAP, NfL, NSE and t-tau and brain imaging showing neuronal injury. The primary endpoint of the Xe-HYPOTHECA trial was the ischaemic brain white matter injury as assessed by FA, from diffusion tensor MRI.

Detailed information on the study recruitment, inclusion and exclusion criteria, and primary outcomes has been reported in a previous publication and its supplementary study protocol.^24^

### Magnetic resonance acquisition and imaging analysis

Patients were scheduled to undergo brain MRI within 16 h after rewarming; ranging from 36 to 52 h after OHCA by using a Siemens Magnetom Verio 3T scanner (Siemens Medical Solutions) with a 12-element Head Matrix coil at both centres with identical imaging protocols. DTI data were acquired using a fat-saturated diffusion-weighted spin-echo echo planar imaging (SE-EPI) sequence with 20 diffusion encoding directions (2 averages, *b*-values 0 and 1000 s/m^2^) and acquisition parameters of TR 6100 ms, TE 100 ms, FOV 250 × 250 mm^2^ and 3 mm slice thickness. GRAPPA parallel acquisition technique with the acceleration factor of 2 and the Phase partial Fourier technique with setting 6/8 were used to accelerate acquisition and obtain images with 2 × 2 × 3 mm^3^ resolution. A three-dimensional magnetization-prepared rapid gradient-echo sequence was used to obtain high-resolution structural 3D images. Following acquisition parameters were used: TR 1900 ms, TE 2.2 ms, TI 900 ms, FA 9 degrees, bandwidth 200 Hz/Px, FOV 250 × 250 × 176 mm^3^ and acquisition resolution of 1 × 1 × 1 mm^3^. Zero-interpolation filling was applied during image reconstruction so that resulting images had 0.5 × 0.5 × 1 mm resolution.

FSL software library (version 5.0, Analysis Group, FMRIB, Oxford, UK) was used for processing the DTI images, following the tract-based spatial statistics processing.^26,27^ The observer-independent and hypothesis-free method can spatially locate group differences in the DTI data. The mean FA, AD, RD and MD values of white matter were calculated as a mean value of all the voxels in the skeleton.

Each subject’s brain-extracted high-resolution 3DT1 images were then segmented into grey matter, white matter and cerebrospinal-fluid regions. The segmented grey matter fraction was then used to create masks for the cortical grey matter and the basal ganglia. Using these masks, MD values of the cortical grey matter and the basal ganglia were measured. All the tools used for this process (FNIRT, FLIRT, FAST and fslstats) are part of the FMRIB Software Library v5.0.^26^

### Biochemical analysis

Blood samples for GFAP, NfL, NSE and t-tau were collected at four timepoints (T), T0 at ∼0 h, T24 at ∼24 h, T48 at ∼48 h and T72 at ∼72 h after the insult. Biomarker measurements were performed as previously published.^28^

### Statistical analysis

The sample size of 110 patients was based on a power analysis of the FA values from brain MRI, i.e. the primary endpoint of the Xe-HYPOTHECA trial.^24^ Between-group differences in continuous baseline characteristics were compared with two-sample *t*-test and Mann–Whitney U-test. The chi-square test and Fisher’s exact test were used to test group differences in categorical baseline characteristics.

The correlations between biomarkers levels at assessed time points in the control group and in the xenon group were calculated with the Spearman correlation coefficient. The differences in biomarker levels between groups were analysed with analysis of covariance after adjustment for age, sex and site. Log-transformed values were used in analyses due to positively skewed distributions. Results are presented as adjusted geometric means (95% confidence interval) and group differences as adjusted geometric mean ratios (95% CI).

Brain white and grey matter microstructural injury metrics between-group comparisons were done with two-sample *t*-test and analysis of covariance after adjustment for age, sex and site.

The correlations between biomarker levels and brain white and grey matter microstructural injury metrics were calculated with Spearman partial correlation coefficients after adjustment for age, sex and site. Fisher Z-transformation tests were used to compare the NfL–FA partial correlations between xenon and control groups at different time points. Age and sex were included due to their known associations with both DTI metrics and biomarker concentrations, while study site was included to account for potential centre-level variability in imaging (i.e. two MRI scanners although from the same manufacturer) and sampling procedures.^29-33^ Finally, inclusion of these covariates improves comparability with prior analyses.^13^

To account for the longitudinal nature of the biomarker data, we performed repeated-measures analyses of covariance. Biomarker concentrations were log-transformed due to right-skewed distributions. Models included fixed effects for time, treatment group (xenon versus control) and their interaction (group × time), and were adjusted for age, sex and study site. An unstructured covariance matrix was selected based on Schwarz’s Bayesian Criterion (BIC). Restricted maximum likelihood estimation was used to obtain unbiased and efficient parameter estimates for data with missing values in follow-up measurements. MRI DTI metrics were obtained at a single time point per patient and were therefore not included in longitudinal trajectory modelling. Formal adjustment for multiple comparisons was not performed due to the exploratory nature of the analyses. Statistical analyses were performed using SAS for Windows, version 9.4 (SAS Institute Inc., Cary, NC, USA). Two-sided *P*-values of less than 0.05 were considered statistically significant.

## Results

A total of 224 patients were screened for eligibility, of whom 110 (aged 24 to 76 years) were enrolled in the original study and randomly assigned in equal numbers to the xenon group (*n* = 55) and the control group (*n* = 55). Patients from the original study whose blood samples were available and measured at the 0 h, 24, 48, and 72 h time points for biochemical analyses were included in this study. Blood samples were available for analysis of GFAP, NfL, NSE and t-tau at the above points in 52 patients in the xenon group and 53 patients in the control group. There were no differences in the baseline characteristics between the groups. Demographic data are shown in Table 1. MRI data were obtained for the correlation analysis from 48 patients in the xenon group and from 48 patients in the control group (Flow chart; Fig. 1). Spearman correlations between biomarker levels at assessed time points in the xenon group and in the control group are presented in Supplementary Tables 1 and 2, respectively. Results of DTI in the complete case population are shown in Supplementary Table 3.

**Figure 1:**
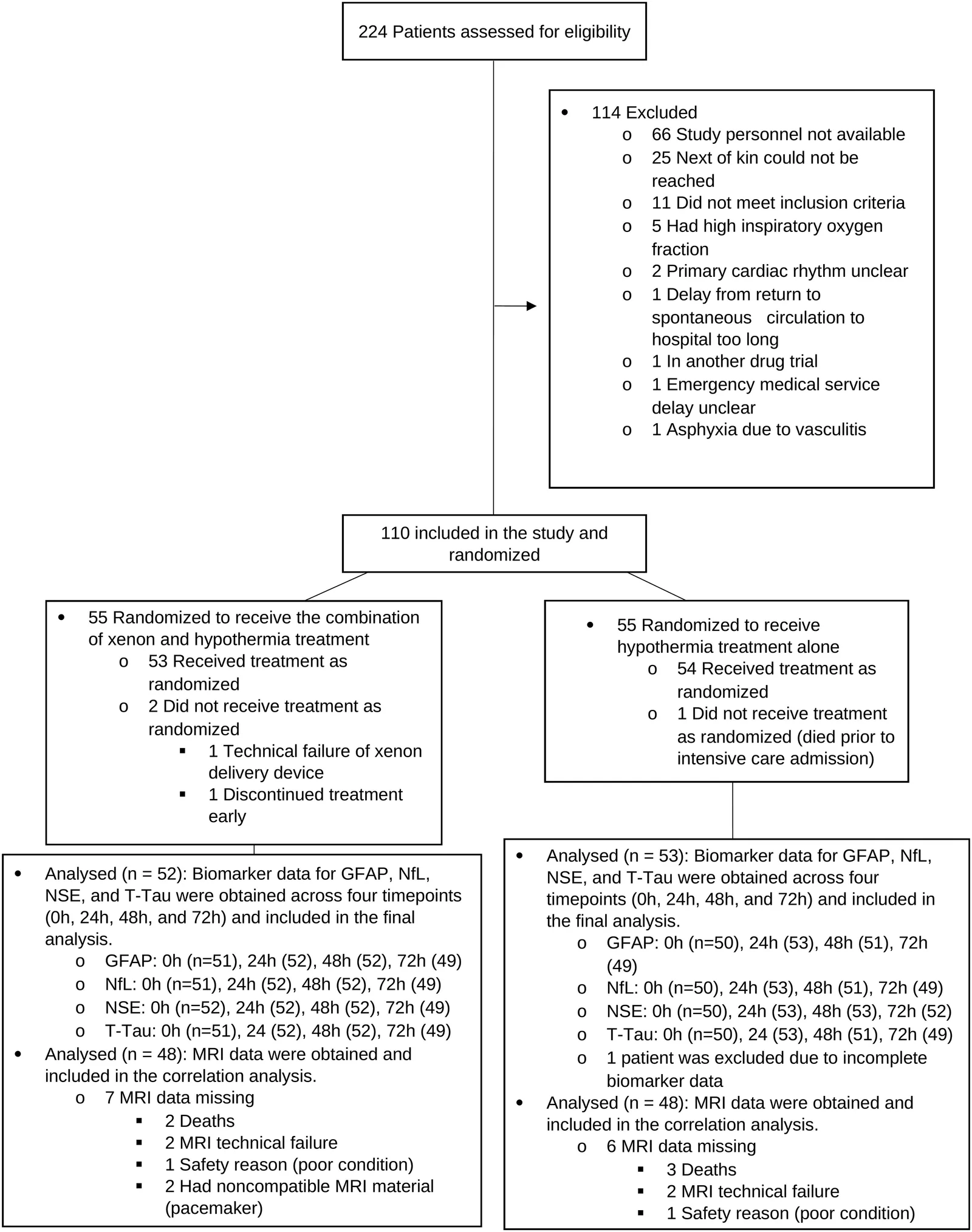
Flow of participants in the study correlation of blood-based biomarkers with microstructural brain injury in survivors of out-of-hospital cardiac arrest.

**Table 1:** Baseline characteristics of the study groups

Variable		Xenon group (*n* = 52)	Control group (*n* = 53)
Age, mean (SD), years		60.33 (11.58)	59.42 (10.88)
Male sex (%)		38 (71.7)	38 (73.1)
Coronary artery disease (%)		35 (67.3)	39 (73.6)
Hypertension		20 (38.46)	25 (47.2)
Congestive heart failure^a^		6 (11.5)	3 (5.66)
Diabetes		8 (15.4)	7 (13.2)
Asthma or chronic obstructive pulmonary disease		6 (11.5%)	8 (15.1)
Dyslipidaemia		15 (28.9)	21 (39.6)
Smoker		15 (30.0)	21 (40.4)
ST-elevation myocardial infarction		16 (30.8)	19 (35.9)
PCI and new stent		14 (26.9)	19 (35.9)
Earlier stroke		11 (22.9)	13 (26.0)
White matter degeneration	Fazekas 0–1^b^	38 (79.2)	40 (80.0)
	Fazekas 2–3^b^	10 (20.8)	10 (20.00)
Resuscitation details	Bystander resuscitation	36 (69.2)	39 (73.6)
	Delay in EMS, mean (SD), min	8.35 (3.43)	8.75 (3.35)
	ROSC, mean (SD), min	22.04 (7.56)	22.02 (7.04)
	No flow, median (IQR), min^c^	0 (0, 6.00)	0 (0, 0)
Core temperature before cooling, median (IQR), °C		34.90 (34.25, 35.80)	35.40 (34.00, 36.30)
Time from out-of-hospital cardiac arrest to target temperature, median (IQR), min		293.00 (254.50, 340.00)	336.00 (261.00, 395.00)
Cooling rate, median (IQR), °C/h		0.42 (0.28, 0.50)	0.43 (0.22, 0.50)
Time from out-of-hospital cardiac arrest to initiation of xenon, median (IQR), min		251.00 (210.00, 282.00)	

IQR, interquartile range; EMS, emergency medical services; ROSC, return of spontaneous circulation.

^a^Based on patient history and angiographic findings.

^b^A magnetic resonance imaging system of scoring for white matter hyperintensities that are often observed in older persons (65 years) and are considered small vessel disease. A score of 0 indicates no lesions; 1, punctate foci; 2, beginning confluence of foci; and 3, large confluent areas.

^c^Indicates period from cardiac arrest to start of any chest compression by bystander or emergency medical personnel.

### Biomarker levels in patients who received inhaled xenon and controls adjusted for age, sex and site

No statistically significant differences were found between the xenon and control groups in the adjusted geometric mean levels of GFAP, NfL, NSE or t-tau at various time points following OHCA (Table 2). Original values of GFAP, NfL, NSE and t-tau are presented in Supplementary Table 4. The biomarker concentrations are shown as original values with logarithmic scaling applied to the *y*-axis in Fig. 2.

**Figure 2:**
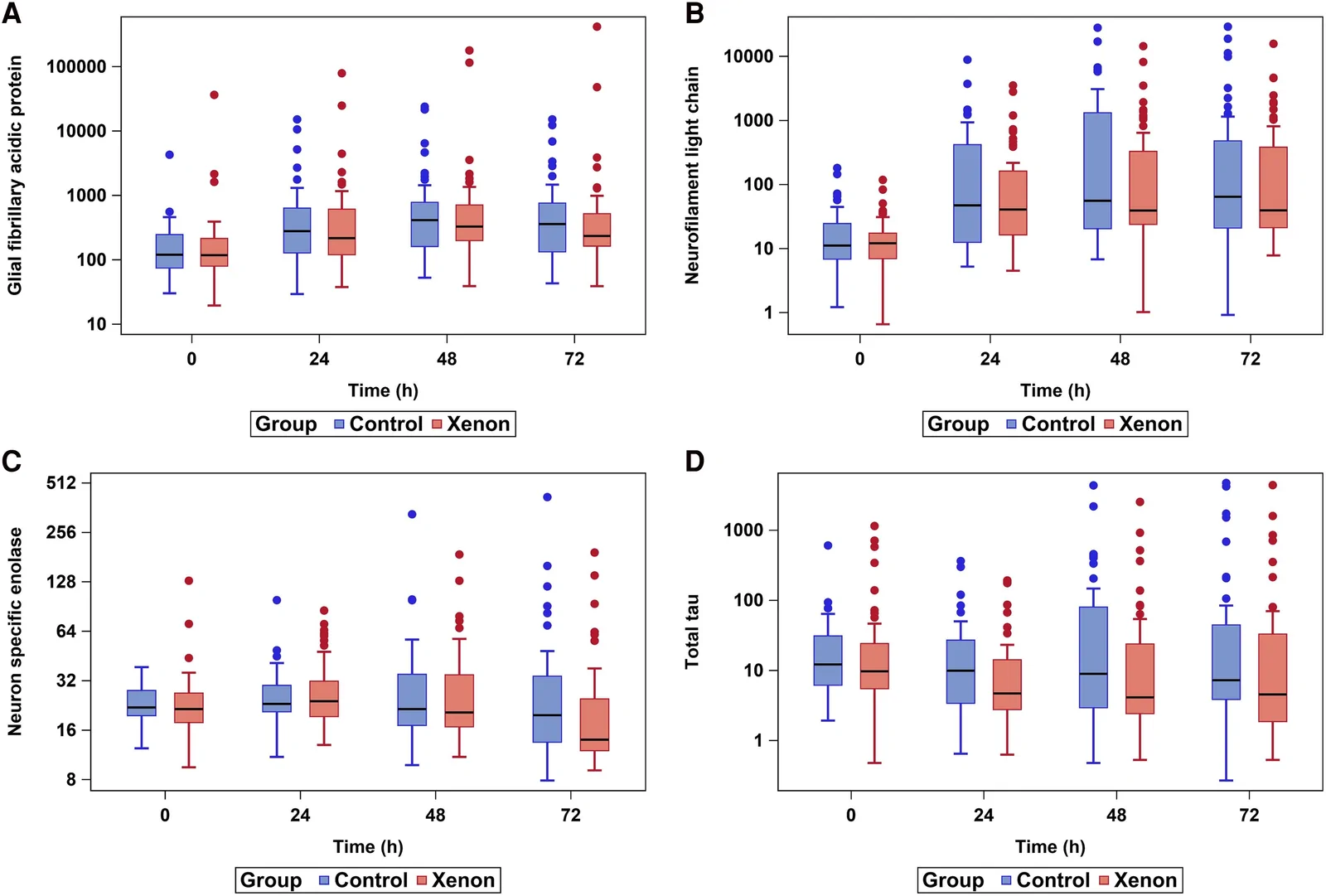
**Biomarker levels in the xenon and control groups at different time points.** (**A**) GFAP, glial fibrillary acidic protein; (**B**) NfL, neurofilament light chain; (**C**) NSE, neuron-specific enolase; (**D**) t-tau, total-tau. Biomarker concentrations are shown as original values with logarithmic scaling applied to the *y*-axis (log_2_ for NSE and log_10_ for the other biomarkers). Box represents lower and upper quartiles, horizontal line in box shows median, whiskers show values that fall within 1.5× interquartile range and points show values that fall outside these limits. No statistically significant differences were found between the xenon and control groups at any time points (analysis of covariance adjusted for age, sex and site). Number of observations was between 49 and 52 in the xenon group and between 49 and 53 in the control group.

**Table 2:** Biomarker geometric mean levels and differences between the xenon and control groups

Biomarker	Adjusted geometric mean (95% CI)				Group difference adjusted geometric mean ratio (95% CI)	*P-*value (adjusted geometric mean ratio)
	Xenon group		Control group			
GFAP		*n*		*n*		
0 h	135.85 (101.62–181.61)	51	137.83 (103.62–183.33)	50	0.99 (0.67–1.44)	0.94
24 h	309.79 (211.57–453.60)	52	339.72 (233.49–494.29)	53	0.91 (0.55–1.50)	0.72
48 h	410.16 (274.73–612.35)	52	439.73 (292.47–661.14)	51	0.93 (0.55–1.58)	0.79
72 h	361.16 (237.99–548.07)	49	375.03 (245.53–572.82)	49	0.96 (0.55–1.68)	0.89
NfL						
0 h	12.28 (9.54–15.80)	51	14.04 (10.96–17.99)	50	0.87 (0.63–1.22)	0.42
24 h	54.94 (33.00–91.46)	52	73.88 (44.75–121.95)	53	0.74 (0.38–1.45)	0.38
48 h	80.63 (43.44–149.68)	52	125.26 (66.75–235.07)	51	0.64 (0.29–1.45)	0.29
72h	90.12 (47.86–169.71)	49	110.63 (58.17–210.40)	49	0.81 (0.35–1.89)	0.63
NSE						
0 h	22.20 (19.98–24.67)	52	22.00 (19.83–24.41)	53	1.01 (0.88–1.16)	0.90
24 h	26.67 (23.61–30.12)	52	24.88 (22.07–28.04)	53	1.07 (0.91–1.26)	0.39
48 h	24.96 (20.67–30.14)	52	26.02 (21.62–31.32)	53	0.96 (0.75–1.23)	0.74
72 h	19.14 (15.23–24.06)	49	23.30 (18.65–29.10)	52	0.82 (0.61–1.11)	0.20
Total-tau						
0 h	16.11 (10.62–24.43)	51	15.48 (10.28–23.30)	50	1.04 (0.60–1.80)	0.88
24 h	6.96 (4.62–10.48)	52	10.40 (6.95–15.56)	53	0.67 (0.39–1.15)	0.14
48 h	8.16 (4.59–14.49)	52	15.38 (8.57–27.60)	51	0.53 (0.24–1.13)	0.10
72 h	10.37 (5.38–19.97)	49	15.24 (7.82–29.66)	49	0.68 (0.28–1.63)	0.38

The adjusted geometric mean ratio for the group difference was calculated as the adjusted geometric mean in the xenon group divided by that in the control group. Values were estimated using an analysis of covariance model adjusted for age, sex and study site.

GFAP, glial fibrillary acidic protein; NSE, neuron-specific enolase; NfL, neurofilament light chain; T-tau, total-tau.

### Longitudinal analysis of biomarker trajectories

In repeated-measures analyses adjusted for age, sex and site, GFAP and NfL increased significantly from baseline at all follow-up time points in both groups, whereas NSE and total-tau showed more modest and time-dependent changes (Supplementary Table 5). No statistically significant group × time interactions were observed for GFAP, NfL, NSE or total-tau, indicating that biomarker trajectories over time did not differ significantly between the xenon and control groups (Supplementary Table 5).

### Adjusted partial correlation between biomarker levels and white matter injury in xenon-treated and control patients

#### Neurofilament light chain

In the xenon group, there was no significant partial correlation between NfL levels and microstructural white matter injury metrics at any time point. However, in the control group, a significant negative partial correlation with FA was observed with NfL at 24, 48 and 72 h (ρ = −0.31 at 24 h, −0.34 at 48 h and −0.37 at 72 h; *P* < 0.05). The NfL–FA partial correlations did not differ significantly between the xenon and control groups at 0 h (ρ = −0.28 versus ρ = −0.02, *P* = 0.21), but the between-group difference increased over time, becoming significant only at 72 h (ρ = −0.37 versus ρ = 0.14, *P* = 0.016).

In the xenon group, xenon was initiated before the 0 h blood sample in 18 of 52 patients (34.6%). Of these, 17 had an available FA value and were included in the correlation analysis. In a *post hoc* sensitivity analysis, NfL–FA partial correlations were calculated separately for patients sampled before versus after xenon initiation. No significant correlations were found at any timepoint in either subgroup, including among those sampled before xenon commenced (ρ = −0.19 at 0 h, *P* = 0.49).

#### Glial fibrillary acidic protein

In the xenon group, no significant partial correlation was observed between GFAP levels and white matter microstructural injury metrics at any time point. Likewise, no significant partial correlation between GFAP levels and FA was observed in the control group.

#### Neuron-specific enolase

In the xenon group, there was no significant partial correlation between the NSE levels and the white matter microstructural injury metrics at any time point. In the control group, NSE levels showed a significant negative partial correlation with FA at 48 h (ρ = −0.30, *P* = 0.04) and 72 h (ρ = −0.35; *P* = 0.02).

#### Total-tau

In the xenon group, there was no significant partial correlation between the t-tau levels and white matter microstructural injury metrics at any time point. In the control group, a significant partial correlation between the t-tau levels and FA was observed at the 72 h timepoint (ρ = −0.30; *P* < 0.05).

Partial correlations between adjusted biomarker levels and brain white matter microstructural injury metrics are shown for the xenon group in Table 3 and for the control group in Table 4. Unadjusted correlations between biomarker levels and brain white matter microstructural metrics are shown for the xenon group in Supplementary Table 6 and for the control group in Supplementary Table 7.

**Table 3:** Spearman partial correlations between biomarkers levels and diffusion tensor magnetic resonance imaging brain microstructure injury metrics in the xenon group; adjusted for age, sex and site

		GFAP					NfL			
		FA	AD	RD	MD		FA	AD	RD	MD
	*n*					*n*				
0 h	47	−0.08	0.27	0.15	0.22	47	−0.02	−0.03	−0.02	−0.00
		*P* = 0.60	*P* = 0.07	*P* = 0.33	*P* = 0.16		*P* = 0.89	*P* = 0.84	*P* = 0.92	*P* = 0.98
24 h	48	0.11	−0.01	−0.13	−0.11	48	0.04	−0.17	−0.16	−0.18
		*P* = 0.48	*P* = 0.97	*P* = 0.38	*P* = 0.48		*P* = 0.80	*P* = 0.26	*P* = 0.29	*P* = 0.24
48h	48	0.03	0.06	−0.06	−0.04	48	−0.01	−0.14	−0.11	−0.13
		*P* = 0.84	*P* = 0.69	*P* = 0.69	*P* = 0.80		*P* = 0.96	*P* = 0.37	*P* = 0.46	*P* = 0.38
72 h	45	0.03	−0.03	−0.08	−0.09	45	0.14	−0.22	−0.22	−0.25
		*P* = 0.85	*P* = 0.85	*P* = 0.59	*P* = 0.59		*P* = 0.38	*P* = 0.16	*P* = 0.16	*P* = 0.11
		**NSE**					**T-tau**			
	*n*	FA	AD	RD	MD	*n*	FA	AD	RD	MD
0 h	48	0.01	−0.09	−0.04	−0.08	47	−0.12	0.18	0.12	0.13
		*P* = 0.96	*P* = 0.57	*P* = 0.78	*P* = 0.59		*P* = 0.43	*P* = 0.25	*P* = 0.42	*P* = 0.39
24 h	48	0.01	−0.05	−0.04	−0.06	48	0.01	0.12	−0.02	0.02
		*P* = 0.93	*P* = 0.73	*P* = 0.81	*P* = 0.68		*P* = 0.96	*P* = 0.43	*P* = 0.90	*P* = 0.88
48h	48	0.09	−0.12	−0.14	−0.13	48	−0.13	0.06	0.04	0.03
		*P* = 0.54	*P* = 0.42	*P* = 0.36	*P* = 0.38		*P* = 0.41	*P* = 0.68	*P* = 0.77	*P* = 0.86
72 h	47	0.05	−0.13	−0.11	−0.14	45	0.05	−0.13	−0.13	−0.16
		*P* = 0.75	*P* = 0.40	*P* = 0.48	*P* = 0.38		*P* = 0.75	*P* = 0.40	*P* = 0.41	*P* = 0.31

In each cell, the top number is a Spearman correlation coefficient, and the number below is a *P*-value; statistically significant correlations are marked by bold print.

GFAP, glial fibrillary acidic protein; NSE, neuron-specific enolase, NfL, neurofilament light chain; T-tau, total-tau; FA, fractional anisotropy; AD, axial diffusivity; RD, radial diffusivity; MD, mean diffusivity.

**Table 4:** Spearman partial correlations between biomarker levels and diffusion tensor magnetic resonance imaging brain microstructure injury metrics in the control group; adjusted for age, sex and site

		GFAP					NfL			
	*n*	FA	AD	RD	MD	*n*	FA	AD	RD	MD
0 h	46	−0.22	0.05	0.14	0.12	46	−0.28	0.15	0.20	0.19
		*P* = 0.15	*P* = 0.73	*P* = 0.38	*P* = 0.43		*P* = 0.07	*P* = 0.35	*P* = 0.20	*P* = 0.22
24 h	48	−0.20	−0.005	0.06	0.06	48	−0.31	0.06	0.16	0.16
		*P* = 0.19	*P* = 0.98	*P* = 0.69	*P* = 0.67		***P*** **=** **0.04**	*P* = 0.68	*P* = 0.28	*P* = 0.29
48 h	46	−0.26	0.05	0.12	0.12	46	−0.34	0.10	0.19	0.18
		*P* = 0.09	*P* = 0.75	*P* = 0.45	*P* = 0.44		***P*** **=** **0.03**	*P* = 0.54	*P* = 0.22	*P* = 0.24
72 h	45	−0.28	0.10	0.18	0.17	45	−0.37	0.14	0.24	0.22
		*P* = 0.08	*P* = 0.51	*P* = 0.25	*P* = 0.28		***P*** **=** **0.02**	*P* = 0.39	*P* = 0.13	*P* = 0.16
		**NSE**					**T-tau**			
	*n*	FA	AD	RD	MD	*n*	FA	AD	RD	MD
0 h	48	0.28	−0.24	−0.35	−0.31	46	−0.11	−0.09	−0.04	−0.05
		*P* = 0.07	*P* = 0.11	***P*** **=** **0.02**	***P*** **=** **0.04**		*P* = 0.48	*P* = 0.58	*P* = 0.80	*P* = 0.75
24 h	48	−0.13	0.001	0.01	0.02	48	−0.16	−0.01	0.03	0.04
		*P* = 0.41	*P* = 0.99	*P* = 0.94	*P* = 0.90		*P* = 0.29	*P* = 0.94	*P* = 0.86	*P* = 0.81
48 h	48	−0.30	0.02	0.13	0.10	46	−0.24	0.04	0.10	0.10
		***P*** **=** **0.04**	*P* = 0.92	*P* = 0.39	*P* = 0.50		*P* = 0.13	*P* = 0.81	*P* = 0.53	*P* = 0.52
72 h	48	−0.35	−0.03	0.16	0.12	45	−0.34	0.08	0.19	0.18
		***P*** **=** **0.02**	*P* = 0.85	*P* = 0.30	*P* = 0.45		***P*** **=** **0.03**	*P* = 0.63	*P* = 0.22	*P* = 0.26

In each cell, the top number is a Spearman correlation coefficient, and the number below is a *P*-value; statistically significant correlations are marked by bold print.

GFAP, glial fibrillary acidic protein; NfL, neurofilament light chain; NSE, neuron-specific enolase, T-tau, total-tau; FA, fractional anisotropy; AD, axial diffusivity; RD, radial diffusivity; MD, mean diffusivity.

### Adjusted partial correlation between biomarker levels and brain grey matter injury in xenon-treated and control patients

A significant negative correlation was observed between MD of basal ganglia and NfL at 72 h in xenon group (ρ = −0.41, *P* = 0.006), and a negative correlation between t-tau and MD of basal ganglia at 72 h (ρ = −0.34, *P* = 0.03).

The results of diffusion metrics are shown for the xenon group in Table 5 and for the control group in Table 6. Unadjusted correlations between biomarker levels and brain grey matter microstructural metrics are shown for the xenon group in Supplementary Table 8 and for the control group in Supplementary Table 9.

**Table 5:** Spearman partial correlations between biomarker levels and grey matter mean diffusivity in the xenon group; adjusted for age, sex and site

	GFAP			NfL		
	*n*	Cg	Bg	*n*	Cg	Bg
**0 h**	47	0.02	0.06	47	−0.24	−0.19
		*P* = 0.92	*P* = 0.71		*P* = 0.12	*P* = 0.23
**24 h**	48	−0.15	−0.26	48	−0.20	−0.26
		*P* = 0.32	*P* = 0.08		*P* = 0.18	*P* = 0.09
**48 h**	48	−0.04	−0.24	48	−0.17	−0.21
		*P* = 0.79	*P* = 0.11		*P* = 0.26	*P* = 0.17
**72 h**	45	0.18	−0.28	45	−0.27	−0.42
		*P* = 0.35	*P* = 0.07		*P* = 0.08	***P*** **=** **0.006**
	**NSE**			**T-tau**		
	*n*	Cg	Bg	*n*	Cg	Bg
**0 h**	47	−0.10	−0.07	47	−0.08	−0.06
		*P* = 0.52	*P* = 0.63		*P* = 0.60	*P* = 0.69
**24 h**	48	−0.06	−0.10	48	−0.07	−0.90
		*P* = 0.67	*P* = 0.52		*P* = 0.67	*P* = 0.56
**48 h**	48	−0.14	−0.27	48	0.05	−0.12
		*P* = 0.36	*P* = 0.07		*P* = 0.77	*P* = 0.45
**72 h**	45	−0.20	−0.25	45	−0.15	−0.34
		*P* = 0.21	*P* = 0.11		*P* = 0.34	***P*** **=** **0.03**

In each cell the top number is a Spearman correlation coefficient, and the number below is a *P*-value; statistically significant correlations are marked by bold print.

GFAP, glial fibrillary acidic protein; NfL, neurofilament light chain; NSE, neuron-specific enolase; T-tau, total-tau; MD, mean diffusivity; Bg, basal ganglia; Cg, cortical grey matter.

**Table 6:** Spearman partial correlations between biomarker levels and grey matter mean diffusivity in the control group; adjusted for age, sex and site

		GFAP			NfL	
	*n*	Cg	Bg	*n*	Cg	Bg
**0 h**	46	0.01	0.02	46	0.12	0.15
		*P* = 0.94	*P* = 0.89		*P* = 0.44	*P* = 0.33
**24 h**	48	−0.08	−0.08	48	0.10	−0.05
		*P* = 0.61	*P* = 0.59		*P* = 0.50	*P* = 0.74
**48 h**	46	−0.06	0.001	46	0.12	−0.003
		*P* = 0.69	*P* > 0.99		*P* = 0.44	*P* = 0.99
**72 h**	45	0.03	0.01	45	0.19	0.03
		*P* = 0.85	*P* = 0.93		*P* = 0.23	*P* = 0.84
		**NSE**			**T-tau**	
	*n*	Cg	Bg	*n*	Cg	Bg
**0 h**	46	−0.47	−0.16	46	−0.10	0.004
		***P*** **=** **0.002**	*P* = 0.29		*P* = 0.53	*P* = 0.98
**24 h**	48	−0.08	0.02	48	−0.04	−0.04
		*P* = 0.60	*P* = 0.88		*P* = 0.79	*P* = 0.81
**48 h**	46	0.02	−0.04	46	0.02	0.001
		*P* = 0.91	*P* = 0.78		*P* = 0.92	*P* > 0.99
**72 h**	45	0.03	0.04	45	0.07	0.05
		*P* = 0.83	*P* = 0.78		*P* = 0.64	*P* = 0.77

In each cell the top number is a Spearman correlation coefficient, and the number below is a *P*-value; statistically significant correlations are marked by bold print.

GFAP, glial fibrillary acidic protein; NfL, neurofilament light chain; NSE, neuron-specific enolase; T-tau, total-tau; MD, mean diffusivity; Bg, basal ganglia; Cg, cortical grey matter.

## Discussion

The primary finding of this study was that NfL levels did not correlate with white matter injury, assessed by FA, in the xenon group following OHCA. Fractional anisotropy was the primary endpoint of the Xe-HYPOTHECA trial. In contrast, statistically significant negative partial correlations between FA and NfL levels were observed in the control group across all three time points. A formal between-group comparison of the NfL–FA correlations reached statistical significance at 72 h, indicating a difference between the treatment groups. Whether this reflects a drug-related effect remains uncertain, and our analyses should be considered exploratory. In addition, GFAP did not show any partial correlation with DTI measures of white matter injury, whereas NSE and t-tau demonstrated the ability to reflect disruption of white matter microintegrity in the control group, albeit to a lesser extent than NfL. There were no consistent partial correlations between cortical or basal ganglia grey matter MD and blood biomarkers in the control group or in the xenon group.

Elevated levels of blood-based biomarker NSE post-OHCA have been associated with both poor neurological outcome and hypoxic-ischaemic encephalopathy findings on head CT.^34^ Most studies on biomarkers following OHCA aim to distinguish between patients with favourable or unfavourable outcomes at a certain time point.^35,36^ To the best of our knowledge, no previous studies have examined how different blood-based biomarkers reflect white and grey matter damage detected by DTI at the early phase following OHCA.

Biomarkers differ from each other based on their origin, thus providing insights into possible brain damage from different perspectives. NSE is released from neurons and predominantly found in the grey matter of the brain,^37^ but it may not be specific to brain injuries, as it is expressed in tissues outside the brain. In addition, haemolysis affects NSE results.^34^ T-tau is a microtubule-associated protein expressed in axons and axon terminals.^38,39^ NfL is a component of neuronal axonal support structure, predominantly expressed by large, myelinated axons within the white matter.^40^ NfL is an indicator of axonal injury in several neurological diseases.^41^ GFAP is an intermediate-filament component of astrocytic cytoskeleton mainly expressed in the central nervous system but also in non-myelinating Schwann cells and enteric glial cells.^42^ NSE is the only blood biomarker currently recommended in post-cardiac arrest treatment guidelines of European Resuscitation Council (ERC) and European Society of Intensive Care Medicine (ESICM).^34^

This study assessed the integrity of white matter microstructure primarily using FA as a key metric. Fractional anisotropy, derived from DTI, is a metric that mirrors the attributes of white matter like fibre density, orderly alignment, myelination and axon width. Decreased FA values in ischaemic white matter likely indicate a mixture of myelin deterioration, axonal decay, and swelling, all of which lead to decreased directional organization of diffusion within white matter tracts. Findings from radiologic studies suggest an increased susceptibility of white matter to ischaemic damage, and there appears to be a correlation between the severity of white matter injury and patient outcome after cardiac arrest.^24,43,44^ DTI potentially offers an added value in predicting outcomes following a cardiac arrest. Studies regarding early DTI imaging and correlation with neurologic recovery are few; however, two studies showed that DTI, conducted roughly two weeks post-cardiac arrest, has a high predictive accuracy to predict unfavourable neurological outcome, defined as dependency or death, with a specificity of 100% and a sensitivity of 90% to 94%.^44,45^ The reduced predictive value of FA during the early sub-acute phase could be attributed to the progressive nature of white matter damage after OHCA.^46^

Conventional blood-based biomarkers such as NSE have been extensively studied in the context of OHCA, however, studies combining biomarkers and DTI imaging in OHCA to our knowledge do not exist. Studies on the blood levels of biomarkers and DTI exist in patients with TBI. In a study including patients with mild TBI, Hossain *et al*.^12^ reported a negative correlation between plasma NfL levels as measured within 24 h of admission and DTI imaging for diffuse axonal injury (all mild TBI patients with a correlation coefficient (ρ) of −0.323 and in CT-positive patients −0.389) in patients with incomplete recovery, whereas correlations were absent in the complete recovery subgroup. Hossains’ MRI was done at 90 days after TBI. Similarly, Castaño-Leon *et al*.^47^ reported significant correlations with DTI imaging and the biomarkers NfL (ρ = −0.471, *P* < 0.001), GFAP (ρ = −0.369, *P* = 0.002) and t-tau (ρ = −0.313, *P* = 0.008) measured within 24 h of the traumatic brain injury in the whole corpus callosum.^47^ Imaging was performed as soon as possible, based on the patient’s medical condition, with a median of 13 days [interquartile range (IQR) 13, range 1–38 days] post-injury. In contrast with current MRI in the early phase, both Hossains’ and Castaño-Leons MRIs were performed later, possibly allowing for more time for the injury-related changes to evolve. This temporal difference as well as pathophysiological differences might partly explain the difference in statistical significance between biomarker correlations and FA observed in their studies versus ours.

In TBI, the axonal injury largely occurs during the blow to the head, and the glial response begins immediately––both develop progressively over hours to days, often with early disruption of axonal transport and collapse of the cytoskeleton.^48,49^ This process leads to the release of biomarkers such as GFAP, NfL and t-tau in the bloodstream.^50^ In contrast, global ischaemia leads to delayed and spatially heterogeneous white matter injury, with early phase changes often caused by energy failure and ion imbalance rather than structural disruption of the cytoskeleton.^3^ Therefore, the release kinetics of these biomarkers in peripheral blood may not align temporally with the DTI changes in OHCA, especially in the acute phase, resulting in weak or no correlations.

In the present study, NfL levels were significantly negatively partially correlated with the FA values at time points 24, 48 and 72 h in the control group, supporting the benefit of NfL in conjunction with DTI imaging in OHCA patients. However, in the xenon group, this correlation was absent. Whether this absent correlation reflects a drug-related effect or pre-existing differences between groups warrants consideration. We performed a *post hoc* sensitivity analysis examining NfL–FA correlations separately in patients who provided their 0 h sample before xenon initiation and those sampled afterwards. We found no significant correlations at any time point in either subgroup. In addition, the 0 h NfL–FA correlation was also non-significant in the control group in the primary analysis, further supporting the interpretation that the absence of correlation at this early timepoint reflects the temporal dynamics of biomarker release rather than a group-specific effect. Moreover, comparing the NfL–FA partial correlations between the xenon and control groups showed no significant difference at 0 h (*P* = 0.21), with progressive divergence reaching significance only at 72 h (*P* = 0.016). This temporal divergence, with the between-group difference, may partly reflect the known slow kinetics of NfL. In experimental traumatic brain injury, blood NfL peaks within 1–3 days after injury, with an even more delayed peak of 10–21 days in clinical human studies,^51,52^ indicating that measurements within the first 24–48 h may not yet fully reflect cumulative axonal injury. As NfL levels increase towards the peak, the difference in NfL-FA correlations between xenon-treated and control patients may become increasingly detectable, consistent with the difference observed at 72 h. This interpretation remains uncertain and we consider these findings exploratory given the small subgroup sizes, and further studies with larger sample sizes are needed to confirm whether this pattern represents a true drug-related effect.

NSE levels showed a significant negative partial correlation with FA only at 48 and 72 h. This finding supports current guideline recommendations suggesting its diagnostic utility only after 48 h post-OHCA, even when combined with advanced MR imaging techniques such as DTI. Similarly, t-tau levels in the control group exhibited a negative partial correlation with FA values only at 72 h, suggesting its usage for diagnostic purposes in conjunction with DTI only beyond this time point.

GFAP levels demonstrated a similar pattern of negative partial correlations with FA in the control group as observed with NfL. However, none of the partial correlations reached statistical significance. This discrepancy may be related to differing origins of the biomarkers. NfL, a component of the neuronal axonal cytoskeleton, is predominantly localized in white matter.^40^ Conversely, GFAP is expressed in astrocytes, which are distributed across both white and grey matter regions.^42^

Notably, the absence of significant partial correlations in the xenon group suggests that the relationship between circulating biomarkers and white matter microstructural injury may be altered following xenon administration. Rather than indicating a lack of brain injury, this finding may reflect a treatment-related modulation of the biomarker–DTI relationship, whereby these biomarkers are less sensitive to microstructural white matter changes detected by DTI in xenon-treated patients.

In addition, a greater number of associations were observed in unadjusted analyses, several of which were attenuated or no longer present after adjustment for age, sex, and study site. This indicates that part of the observed associations may be explained by these covariates, underscoring the importance of adjusted analyses when interpreting biomarker–imaging relationships. This likely reflects the known influence of age on both DTI metrics and circulating biomarker concentrations, the potential contribution of sex-related differences, and centre-level variability in imaging acquisition and sampling procedures; adjustment for these factors also improves comparability with prior studies.^7,29-33^ Further studies are needed to determine whether similar modulation or attenuation of biomarker correlations is observed with other neuroprotective or neuro-modifying interventions.

In our earlier study,^24^ the primary finding was that xenon demonstrated neuroprotective properties, primarily by attenuating damage to white matter microstructure—an effect largely attributed to reduced demyelination. Radial diffusivity was shown to be the main factor influencing the lower FA values in the control group. The current findings provide complementary evidence: in the control group, adjusted NfL levels showed a significant partial correlation with FA in white matter, indicating a relationship between axonal injury and microstructural disruption (Table 4). Notably, no significant partial correlation was observed in the xenon group. Together with the significant between-group difference in NfL-FA correlations at 72 h, this finding suggests that xenon may mitigate white matter injury. FA is sensitive to myelin and axonal integrity and can decrease in the presence of demyelination.^53^ One potential mechanism involves NMDA receptors, which are expressed in the myelin of white matter oligodendrocytes and become activated during ischaemic events. Xenon acts as an NMDA receptor antagonist by competitively inhibiting the glycine binding site.^15,17^

This study found no consistent partial correlation between grey matter MD and blood biomarkers, likely due to the differing temporal behaviour of MD in grey matter compared to FA in white matter during the early phase following a global ischaemic event. It is well established that during the early stages of ischaemia, cytotoxic oedema in grey matter is characterized by a decrease in MD values. Subsequently, MD values typically return to baseline in a process known as pseudo normalization, followed by an increase as the ischaemic lesion progresses. In contrast, FA values decline and remain low as the lesion evolves from the acute phase (<24 h) to the sub-acute phase (3–5 days) and ultimately to the chronic stage.^54,55^ The exact timing of pseudo normalization remains difficult to determine, as it varies between individuals and is influenced by factors such as the spatial heterogeneity within the ischaemic lesion.^56^

Newcombe *et al*.^13^ previously demonstrated that in patients with TBI, elevated MD in both deep and cortical grey matter, as well as white matter, was positively associated with increased levels of GFAP and NfL.^13^ Their MRI scans were performed six months post-injury, when final tissue damage was evident and characterized by high MD values. In contrast, in the current study, patients were scanned at a median of 53 h post-OHCA (interquartile range: 47–64 h), a period during which both downward and upward changes in grey matter MD are likely occurring. This temporal variability likely disrupts correlation patterns, as some patients may be in the downward phase while others are in the upward phase relative to biomarker sampling times.

The geometric mean biomarker levels of NfL, GFAP, NSE and t-tau did not reach statistical significance between the xenon and control groups. This lack of significance is likely attributable to limited statistical power. Notably, the median values of NfL and t-tau were consistently lower in the xenon group at all post-baseline time points, although these differences did not reach statistical significance. The longitudinal repeated-measures analysis was consistent with the main findings, with the strongest (albeit non-significant) group × time signal observed for t-Tau and a similar directional pattern for NfL. These findings may suggest a drug-related modulation of biomarker–DTI relationships, but should be interpreted cautiously given the sample size. It is important to emphasize that the original Xe-HYPOTHECA trial was not powered to detect differences in biomarker levels. Its primary aim was to evaluate changes in FA between the control and xenon groups. Further research with larger sample size and a protocol specifically designed for biomarker analysis is needed to detect the potential neuroprotective effects of xenon.

These results are in line with previous studies indicating that blood-based biomarkers show promise in the diagnostics of patients who are resuscitated after an OHCA. Their use may improve early neurological assessment, guide the intensity of care and support communication with families and caregivers during critical decisions in the ICU. In future, this may also be valuable in resource-limited settings such as rural hospitals or emergency settings, where rapid biomarker results can help triage and identify patients with the potential for meaningful neurological recovery. While these biomarkers provide objective evidence of hypoxic-ischaemic brain injury, they should be interpreted as complementary tools alongside clinical examination findings, imaging findings, and other prognostic indicators (such as time to return of spontaneous circulation and patient age).

## Limitations

One of the key limitations of this study is the relatively small cohort size, which may affect the generalizability of the findings. Additionally, due to the randomized controlled trial (RCT) design, the cohort may not be fully representative of all OHCA patients, as strict inclusion and exclusion criteria were applied. Furthermore, the small patient population limited the ability to detect differences in biomarker levels between groups.

However, a notable strength of this study is the precise characterization of patients within the framework of the original RCT. This ensures a well-defined study population, allowing for a more controlled analysis of the investigated outcomes. Last, this study was not designed or powered for outcome prediction and focuses on biomarker–DTI associations.

## Conclusion

The primary Xe-HYPOTHECA trial showed that inhaled xenon was associated with less white matter damage in cardiac arrest survivors compared with controls, as measured by FA. In the present study, several blood-based biomarkers of brain damage, particularly NfL, NSE and t-tau, showed significant negative partial correlations with FA in the control group, whereas no corresponding correlations were found in the xenon group. A formal between-group comparison of the NfL–FA correlations indicated a difference between the treatment groups, reaching statistical significance at 72 h. These exploratory findings suggest that xenon may modify the relationship between axonal injury and white matter microstructure after OHCA, although confirmation in larger studies is needed. The findings also support the potential role of blood-based biomarkers in evaluating brain injury in patients resuscitated after OHCA.

## Supplementary Material

fcag378_Supplementary_Data

## Data Availability

The datasets used and/or analysed during the current study are available from the corresponding author on reasonable request. Only open access MRI post-processing tools were used.

## Appendix

### The Xe-HYPOTHECA Working Group

Juhani Airaksinen, Juha Grönlund, Veli-Pekka Harjola, Outi Inkinen, Kirsi Korpi, Jussi Niiranen, Eija Nukarinen, Klaus T. Olkkola, Mikko Pietilä, Antti Saraste, Päivi Silvasti, Marjut Varpula and Johanna Wennervirta.
