# Supplementary material for: Correlation of blood biomarkers with microstructural brain injury in survivors of out-of-hospital cardiac arrest
Source: Brain Commun. 2026 Oct 8;8(5):fcag378. doi: 10.1093/braincomms/fcag378 (PMC13673109; doi:10.1093/braincomms/fcag378)
Supplement: Supplementary file 1 [file fcag378_supplementary_data.docx]

**Supplementary Table 1.** Spearman correlations between biomarkers levels at assessed time points in the xenon group

|  | **GFAP 0 h**  **(n=51)** | **NfL**  **0 h**  **(n=51)** | **NSE**  **0 h**  **(n=51)** | **T-tau**  **0 h**  **(n=51)** | **GFAP 24 h**  **(n=52)** | **NfL**  **24 h**  **(n=52)** | **NSE**  **24 h**  **(n=52)** | **T-tau 24 h**  **(n=52)** | **GFAP 48 h**  **(n=52)** | **NfL**  **48 h**  **(n=52)** | **NSE**  **48 h**  **(n=52)** | **T-tau 48 h**  **(n=52)** | **GFAP 72 h**  **(n=49)** | **NfL**  **72 h**  **(n=49)** | **NSE**  **72 h**  **(n=47)** | **T-tau**  **72 h**  **(n=49)** |
| --- | --- | --- | --- | --- | --- | --- | --- | --- | --- | --- | --- | --- | --- | --- | --- | --- |
| **GFAP**  **0 h** | 1 | **0.74**  **p<0.001** | -0.15  p=0.29 | **0.42**  **p=0.002** |  |  |  |  |  |  |  |  |  |  |  |  |
| **NfL**  **0 h** |  | 1 | -0.10  p=0.48 | **0.29**  **p=0.04** |  |  |  |  |  |  |  |  |  |  |  |  |
| **NSE**  **0 h** |  |  | 1 | **0.49**  **p<0.001** |  |  |  |  |  |  |  |  |  |  |  |  |
| **T-tau**  **0 h** |  |  |  | 1 |  |  |  |  |  |  |  |  |  |  |  |  |
| **GFAP 24 h** |  |  |  |  | 1 | **0.41**  **p=0.002** | **0.64**  **p<0.001** | **0.77**  **p<0.001** |  |  |  |  |  |  |  |  |
| **NfL**  **24 h** |  |  |  |  |  | 1 | **0.57**  **p<0.001** | **0.33**  **p=0.02** |  |  |  |  |  |  |  |  |
| **NSE**  **24 h** |  |  |  |  |  |  | 1 | **0.73**  **p<0.001** |  |  |  |  |  |  |  |  |
| **T-tau**  **24 h** |  |  |  |  |  |  |  | 1 |  |  |  |  |  |  |  |  |
| **GFAP 48 h** |  |  |  |  |  |  |  |  | 1 | **0.74**  **p<0.001** | **0.43**  **p=0.001** | **0.79**  **p<0.001** |  |  |  |  |
| **NfL**  **48 h** |  |  |  |  |  |  |  |  |  | 1 | **0.56**  **p<0.001** | **0.77**  **p<0.001** |  |  |  |  |
| **NSE**  **48 h** |  |  |  |  |  |  |  |  |  |  | 1 | **0.66**  **p<0.001** |  |  |  |  |
| **T-tau**  **48 h** |  |  |  |  |  |  |  |  |  |  |  | 1 |  |  |  |  |
| **GFAP 72 h** |  |  |  |  |  |  |  |  |  |  |  |  | 1 | **0.78**  **p<0.001** | **0.41**  **p=0.004** | **0.78**  **p<0.001** |
| **NfL**  **72 h** |  |  |  |  |  |  |  |  |  |  |  |  |  | 1 | **0.52**  **p<0.001** | **0.80**  **p<0.001** |
| **NSE**  **72 h** |  |  |  |  |  |  |  |  |  |  |  |  |  |  | 1 | **0.64**  **p<0.001** |
| **T-tau**  **72 h** |  |  |  |  |  |  |  |  |  |  |  |  |  |  |  | 1 |

In each cell, the top number is a Spearman correlation coefficient, and the number below is a p-value; statistically significant correlations are marked by bold print. GFAP, glial fibrillary acidic protein; NF-L, neurofilament light chain; NSE, neuron specific enolase; T-tau, total tau

**Supplementary Table 2.** Spearman correlations between biomarkers levels at assessed time points in the control group

|  | **GFAP 0 h**  **(n=50)** | **NfL**  **0 h**  **(n=50)** | **NSE 0 h**  **(n=50)** | **T-tau**  **0 h**  **(n=50)** | **GFAP 24 h**  **(n=53)** | **NfL**  **24 h**  **(n=53)** | **NSE**  **24 h**  **(n=53)** | **T-tau 24 h**  **(n=53)** | **GFAP 48 h**  **(n=51)** | **NfL**  **48 h**  **(n=51)** | **NSE**  **48 h**  **(n=51)** | **T-tau 48 h**  **(n=51)** | **GFAP 72 h**  **(n=49)** | **NfL**  **72 h**  **(n=49)** | **NSE**  **72 h**  **(n=49)** | **T-tau 72 h**  **(n=49)** |
| --- | --- | --- | --- | --- | --- | --- | --- | --- | --- | --- | --- | --- | --- | --- | --- | --- |
| **GFAP 0 h** | 1 | **0.71**  **p<0.001** | 0.19  p=0.19 | **0.45**  **p=0.001** |  |  |  |  |  |  |  |  |  |  |  |  |
| **NfL**  **0 h** |  | 1 | 0.19  p=0.20 | **0.54**  **p<0.001** |  |  |  |  |  |  |  |  |  |  |  |  |
| **NSE**  **0 h** |  |  | 1 | 0.26  p=0.07 |  |  |  |  |  |  |  |  |  |  |  |  |
| **T-tau 0 h** |  |  |  | 1 |  |  |  |  |  |  |  |  |  |  |  |  |
| **GFAP 24 h** |  |  |  |  | 1 | **0.83**  **p<0.001** | **0.42**  **p=0.002** | **0.76**  **p<0.001** |  |  |  |  |  |  |  |  |
| **NfL 24 h** |  |  |  |  |  | 1 | **0.83**  **p<0.001** | **0.57**  **p<0.001** |  |  |  |  |  |  |  |  |
| **NSE 24 h** |  |  |  |  |  |  | 1 | **0.61**  **p<0.001** |  |  |  |  |  |  |  |  |
| **T-tau 24 h** |  |  |  |  |  |  |  | 1 |  |  |  |  |  |  |  |  |
| **GFAP 48 h** |  |  |  |  |  |  |  |  | 1 | **0.85**  **p<0.001** | **0.63**  **p<0.001** | **0.80**  **p<0.001** |  |  |  |  |
| **NfL 48 h** |  |  |  |  |  |  |  |  |  | 1 | **0.75**  **p<0.001** | **0.89**  **p<0.001** |  |  |  |  |
| **NSE 48 h** |  |  |  |  |  |  |  |  |  |  | 1 | **0.61**  **p<0.001** |  |  |  |  |
| **T-tau 48 h** |  |  |  |  |  |  |  |  |  |  |  | 1 |  |  |  |  |
| **GFAP 72 h** |  |  |  |  |  |  |  |  |  |  |  |  | 1 | **0.80**  **p<0.001** | **0.52**  **p<0.001** | **0.71**  **p<0.001** |
| **NfL 72 h** |  |  |  |  |  |  |  |  |  |  |  |  |  | 1 | **0.68**  **p<0.001** | **0.86**  **p<0.001** |
| **NSE 72 h** |  |  |  |  |  |  |  |  |  |  |  |  |  |  | 1 | **0.56**  **p<0.001** |
| **T-tau 72h** |  |  |  |  |  |  |  |  |  |  |  |  |  |  |  | 1 |

In each cell, the top number is a Spearman correlation coefficient, and the number below is a p-value; statistically significant correlations are marked by bold print. GFAP, glial fibrillary acidic protein; NF-L, neurofilament light chain; NSE, neuron specific enolase; T-tau, total-tau

| Supplementary Table 3. Results of Diffusion Tensor Magnetic Resonance Imaging in the Complete Case Population | | | | |
| --- | --- | --- | --- | --- |
|  | **Unadjusted Mean (SD)** | | **P Value** | **P Value** |
| White Matter Global Values | **Xenon Group (n = 48)** | **Control Group (n = 48)** | **Unadjusted** | **Adjusted** |
| Fractional anisotrophy | 0.43 (0.03) | 0.42 (0.03) | **0.04** | **0.009** |
| Type of diffusivity |  |  |  |  |
| Axial | 1.19 (0.05) | 1.20 (0.05) | 0.52 | 0.47 |
| Radial | 0.60 (0.05) | 0.62 (0.06) | 0.12 | **0.047** |
| Mean | 0.80 (0.05) | 0.81 (0.06) | 0.19 | 0.10 |
| Grey Matter Global Values | **Xenon Group (n = 48)** | **Control Group (n = 48)** | **Unadjusted** | **Adjusted** |
| MD Cortex | 891.96 (43.13) | 893.42 (50.40) | 0.88 | 0.73 |
| MD BG | 806.92 (69.42) | 810.06 (70.68) | 0.83 | 0.66 |

A two-sample t-test was used to compare unadjusted means between the xenon and control groups. Analysis of covariance was used to compare adjusted means between the groups, with adjustment for age, sex, and study site.

**Supplementary Table 4.** Biomarker levels in the xenon and control groups

|  |  | **Xenon** | | | | | **Control** | | | | |
| --- | --- | --- | --- | --- | --- | --- | --- | --- | --- | --- | --- |
| **Biomarker** | **Timepoint** | **n, samples** | **Median** | **25^th^ Pctl** | **Quartile** | **75^th^ Pctl** | **n, samples** | **Median** | **25^th^ Pctl** | **Quartile** | **75^th^ Pctl** |
| **GFAP** | 0 h | 51 | 118.00 | 79.80 | 135.20 | 215.00 | 50 | 120.00 | 74.60 | 172.40 | 247.00 |
|  | 24 h | 52 | 217.00 | 120.00 | 493.00 | 613.00 | 53 | 279.00 | 128.00 | 508.00 | 636.00 |
|  | 48 h | 52 | 328.50 | 199.00 | 511.50 | 710.50 | 51 | 414.00 | 161.00 | 621.00 | 782.00 |
|  | 72 h | 49 | 235.00 | 163.00 | 358.00 | 521.00 | 49 | 359.00 | 133.00 | 626.00 | 759.00 |
| **Nf-L** | 0 h | 51 | 12.10 | 6.95 | 10.45 | 17.40 | 50 | 11.15 | 11.15 | 17.76 | 24.60 |
|  | 24 h | 52 | 40.65 | 16.35 | 145.65 | 162.00 | 53 | 47.20 | 47.20 | 406.50 | 419.00 |
|  | 48 h | 52 | 39.20 | 23.85 | 305.15 | 329.00 | 51 | 55.70 | 55.70 | 1297.60 | 1318.00 |
|  | 72 h | 49 | 39.50 | 21.20 | 360.80 | 382.00 | 49 | 64.40 | 64.40 | 459.10 | 480.00 |
| **NSE** | 0 h | 52 | 21.50 | 17.80 | 9.10 | 26.90 | 53 | 22.00 | 19.60 | 8.30 | 27.90 |
|  | 24 h | 52 | 24.00 | 19.35 | 12.45 | 31.80 | 53 | 23.10 | 20.70 | 9.30 | 30.00 |
|  | 48 h | 52 | 20.50 | 16.75 | 18.00 | 34.75 | 53 | 21.50 | 17.10 | 17.90 | 35.00 |
|  | 72 h | 49 | 14.00 | 12.00 | 12.80 | 24.80 | 52 | 19.75 | 13.50 | 20.60 | 34.10 |
| **T-tau** | 0 h | 51 | 9.71 | 5.49 | 18.71 | 24.20 | 50 | 12.20 | 6.16 | 24.94 | 31.10 |
|  | 24 h | 52 | 4.71 | 2.77 | 11.43 | 14.20 | 53 | 9.92 | 3.40 | 23.70 | 27.10 |
|  | 48 h | 52 | 4.14 | 2.42 | 4.14 | 23.85 | 51 | 8.95 | 2.94 | 77.16 | 80.10 |
|  | 72 h | 49 | 4.55 | 1.87 | 4.55 | 33.00 | 49 | 7.27 | 3.86 | 40.74 | 44.60 |

GFAP, glial fibrillary acidic protein; Nf-L, neurofilament light chain; NSE, neuron specific enolase; T-tau, total-tau

**Supplementary Table 5.** The differences in the change in biomarker levels between the xenon and control groups

|  |  |  |  |  | Group difference | Group×time |
| --- | --- | --- | --- | --- | --- | --- |
| **Biomarker** | Adjusted Geometric Mean Ratio (95% CI) | | | | Adjusted Geometric | interaction effect |
|  | Control group (n=53) | P-value | Xenon group (n=52) | P-value | Mean Ratio (95% CI) | P-value |
| **GFAP** |  |  |  |  |  |  |
| Change from 0h to 24h | 2.41 (1.88-3.10) | <0.001 | 2.25 (1.75-2.89) | <0.001 | 0.93 (0.65-1.33) | 0.69 |
| Change from 0h to 48h | 3.22 (2.48-4.19) | <0.001 | 3.09 (2.38-4.00) | <0.001 | 0.96 (0.66-1.38) | 0.81 |
| Change from 0h to 72h | 2.77 (2.07-3.70) | <0.001 | 2.61 (1.96-3.48) | <0.001 | 0.94 (0.63-1.42) | 0.78 |
|  |  |  |  |  |  |  |
| **Nfl** |  |  |  |  |  |  |
| Change from 0h to 24h | 5.49 (3.61-8.35) | <0.001 | 4.76 (3.12-7.26) | <0.001 | 0.87 (0.48-1.57) | 0.64 |
| Change from 0h to 48h | 9.34 (5.68-15.36) | <0.001 | 7.27 (4.41-11.99) | <0.001 | 0.78 (0.38-1.57) | 0.48 |
| Change from 0h to 72h | 9.32 (5.52-15.75) | <0.001 | 7.68 (4.54-13.00) | <0.001 | 0.82 (0.39-1.73) | 0.61 |
|  |  |  |  |  |  |  |
| **NSE** |  |  |  |  |  |  |
| Change from 0h to 24h | 1.11 (1.01-1.22) | 0.03 | 1.18 (1.08-1.30) | <0.001 | 1.07 (0.93-1.22) | 0.36 |
| Change from 0h to 48h | 1.15 (0.98-1.36) | 0.08 | 1.11 (0.94-1.30) | 0.22 | 0.96 (0.76-1.21) | 0.73 |
| Change from 0h to 72h | 1.07 (0.87-1.32) | 0.52 | 0.87 (0.70-1.08) | 0.21 | 0.81 (0.60-1.10) | 0.18 |
|  |  |  |  |  |  |  |
| **t-tau** |  |  |  |  |  |  |
| Change from 0h to 24h | 0.69 (0.52-0.91) | 0.009 | 0.49 (0.37-0.64) | <0.001 | 0.70 (0.48-1.04) | 0.08 |
| Change from 0h to 48h | 1.00 (0.63-1.61) | 0.99 | 0.60 (0.37-0.95) | 0.03 | 0.59 (0.30-1.16) | 0.12 |
| Change from 0h to 72h | 1.05 (0.61-1.81) | 0.87 | 0.70 (0.40-1.20) | 0.19 | 0.67 (0.31-1.44) | 0.30 |
|  |  |  |  |  |  |  |
| The adjusted geometric mean ratio was calculated as the adjusted geometric mean at follow-up divided by the adjusted geometric mean at 0 h. The group difference in the adjusted geometric mean ratio was calculated as the adjusted mean ratio for change in the xenon group divided by that in the control group. Values were estimated using contrasts from a repeated-measures analysis of covariance model. The model included the main effects of group and time and the group × time interaction and was adjusted for age, sex, and study site | | | | |  |  |

**Supplementary Table 6.** Spearman correlations between biomarker levels and brain white matter microstructural metrics in the xenon group

|  |  | **GFAP** | | | |  | **NfL** | | | |
| --- | --- | --- | --- | --- | --- | --- | --- | --- | --- | --- |
|  | n | FA | AD | RD | MD | n | FA | AD | RD | MD |
| **0 h** | 47 | -0.25 | 0.17 | 0.27 | 0.27 | 47 | -0.28 | -0.02 | 0.23 | 0.19 |
|  |  | p=0.09 | p=0.27 | p=0.07 | p=0.06 |  | p=0.05 | p=0.87 | p=0.13 | p=0.20 |
| **24 h** | 48 | -0.08 | -0.05 | 0.03 | 0.01 | 48 | 0.08 | -0.18 | -0.04 | -0.08 |
|  |  | p=0.60 | p=0.75 | p=0.83 | p=0.93 |  | p=0.61 | p=0.23 | p=0.77 | p=0.57 |
| **48 h** | 48 | -0.09 | 0.01 | 0.04 | 0.02 | 48 | -0.09 | -0.14 | -0.03 | -0.07 |
|  |  | p=0.54 | p=0.97 | p=0.81 | p=0.92 |  | p=0.55 | p=0.34 | p=0.84 | p=0.63 |
| **72 h** | 45 | -0.11 | -0.06 | 0.04 | 0.00 | 45 | 0.05 | -0.21 | -0.14 | -0.18 |
|  |  | p=0.46 | p=0.71 | p=0.79 | p=1.00 |  | p=0.76 | p=0.16 | p=0.37 | p=0.23 |
|  |  |  |  |  |  |  |  |  |  |  |
|  |  | **NSE** | | | |  | **T-tau** | | | |
|  | n | FA | AD | RD | MD | n | FA | AD | RD | MD |
| **0 h** | 48 | 0.15 | -0.08 | -0.17 | -0.18 | 47 | -0.03 | 0.13 | 0.04 | 0.05 |
|  |  | p=0.31 | p=0.57 | p=0.26 | p=0.23 |  | p=0.85 | p=0.40 | p=0.82 | p=0.73 |
| **24 h** | 48 | 0.04 | -0.05 | -0.05 | -0.06 | 48 | -0.06 | 0.09 | 0.04 | 0.07 |
|  |  | p=0.81 | p=0.76 | p=0.75 | p=0.69 |  | p=0.66 | p=0.55 | p=0.76 | p=0.65 |
| **48 h** | 48 | 0.09 | -0.11 | -0.12 | -0.11 | 48 | -0.18 | 0.03 | 0.09 | 0.06 |
|  |  | p=0.53 | p=0.47 | p=0.40 | p=0.45 |  | p=0.22 | p=0.82 | p=0.53 | p=0.68 |
| **72 h** | 47 | 0.03 | -0.11 | -0.08 | -0.10 | 45 | 0.02 | -0.16 | -0.10 | -0.15 |
|  |  | p=0.85 | p=0.46 | p=0.60 | p=0.49 |  | p=0.88 | p=0.30 | p=0.49 | p=0.32 |

**Supplementary Table 7.** Spearman correlations between biomarker levels and brain white matter microstructural metrics in the control group

|  |  | **GFAP** | | | |  | **NfL** | | | |
| --- | --- | --- | --- | --- | --- | --- | --- | --- | --- | --- |
|  | n | FA | AD | RD | MD | n | FA | AD | RD | MD |
| **0 h** | 46 | **-0.38** | 0.09 | 0.27 | 0.25 | 46 | **-0.43** | 0.19 | 0.33 | 0.31 |
|  |  | **p=0.008** | p=0.55 | p=0.07 | p=0.10 |  | **p=0.003** | p=0.20 | p=0.03 | p=0.03 |
| **24 h** | 48 | **-0.36** | 0.06 | 0.21 | 0.20 | 48 | **-0.42** | 0.12 | 0.28 | 0.27 |
|  |  | **p=0.01** | p=0.71 | p=0.15 | p=0.17 |  | **p=0.003** | p=0.41 | p=0.053 | p=0.06 |
| **48 h** | 46 | **-0.42** | 0.09 | 0.27 | 0.26 | 46 | **-0.43** | 0.12 | 0.28 | 0.27 |
|  |  | **p=0.004** | p=0.53 | p=0.07 | p=0.09 |  | **p=0.003** | p=0.41 | p=0.06 | p=0.07 |
| **72 h** | 45 | **-0.42** | 0.12 | 0.30 | 0.28 | 45 | **-0.45** | 0.13 | 0.30 | 0.28 |
|  |  | **p=0.004** | p=0.45 | **p=0.046** | p=0.07 |  | **p=0.002** | p=0.39 | **p=0.04** | p=0.07 |
|  |  |  |  |  |  |  |  |  |  |  |
|  |  | **NSE** | | | |  | **T-tau** | | | |
|  | n | FA | AD | RD | MD | n | FA | AD | RD | MD |
| **0 h** | 48 | **0.29** | -0.22 | **-0.33** | **-0.30** | 46 | -0.15 | -0.11 | -0.01 | -0.03 |
|  |  | **p=0.045** | p=0.13 | **p=0.02** | **p=0.04** |  | p=0.30 | p=0.46 | p=0.93 | p=0.84 |
| **24 h** | 48 | -0.11 | 0.00 | 0.01 | 0.02 | 48 | -0.22 | -0.02 | 0.07 | 0.07 |
|  |  | p=0.46 | p=0.99 | p=0.95 | p=0.91 |  | p=0.14 | p=0.91 | p=0.62 | p=0.62 |
| **48 h** | 48 | **-0.38** | 0.07 | 0.22 | 0.19 | 46 | **-0.32** | 0.06 | 0.17 | 0.17 |
|  |  | **p=0.008** | p=0.65 | p=0.14 | p=0.20 |  | **p=0.03** | p=0.69 | p=0.25 | p=0.26 |
| **72 h** | 48 | **-0.46** | 0.09 | 0.29 | 0.26 | 45 | **-0.39** | 0.09 | 0.24 | 0.23 |
|  |  | **p=0.001** | p=0.56 | **p=0.04** | p=0.08 |  | **p=0.008** | p=0.55 | p=0.11 | p=0.14 |

**Supplementary Table 8.** Spearman correlations between biomarker levels and brain grey matter microstructural metrics in the xenon group

|  |  | **GFAP** |  |  | **NfL** |  |
| --- | --- | --- | --- | --- | --- | --- |
|  | n | Cg | Bg | n | Cg | Bg |
| **0 h** | 47 | 0.05 | 0.15 | 47 | -0.09 | 0.04 |
|  |  | p=0.73 | p=0.31 |  | p=0.53 | p=0.78 |
| **24 h** | 48 | -0.09 | -0.12 | 48 | -0.16 | -0.16 |
|  |  | p=0.54 | p=0.42 |  | p=0.29 | p=0.29 |
| **48 h** | 48 | -0.03 | -0.12 | 48 | -0.15 | -0.12 |
|  |  | p=0.83 | p=0.42 |  | p=0.32 | p=0.40 |
| **72 h** | 45 | -0.11 | -0.13 | 45 | -0.25 | **-0.30** |
|  |  | p=0.46 | p=0.39 |  | p=0.10 | **p=0.04** |
|  |  |  |  |  |  |  |
|  |  | **NSE** |  |  | **T-tau** |  |
|  | n | Cg | Bg | n | Cg | Bg |
| **0 h** | 47 | -0.15 | -0.18 | 47 | -0.11 | -0.13 |
|  |  | p=0.31 | p=0.23 |  | p=0.44 | p=0.37 |
| **24 h** | 48 | -0.05 | -0.13 | 48 | -0.04 | -0.06 |
|  |  | p=0.72 | p=0.36 |  | p=0.78 | p=0.71 |
| **48 h** | 48 | -0.12 | -0.27 | 48 | 0.05 | -0.07 |
|  |  | p=0.41 | p=0.06 |  | p=0.71 | p=0.65 |
| **72 h** | 45 | -0.18 | -0.22 | 45 | -0.15 | **-0.31** |
|  |  | p=0.23 | p=0.15 |  | p=0.32 | **p=0.04** |

**Supplementary Table 9.** Spearman correlations between biomarker levels and brain grey matter microstructural metrics in the control group

|  |  | **GFAP** |  |  | **NfL** |  |
| --- | --- | --- | --- | --- | --- | --- |
|  | n | Cg | Bg | n | Cg | Bg |
| **0 h** | 46 | 0.12 | -0.01 | 46 | 0.22 | 0.16 |
|  |  | p=0.43 | p=0.94 |  | p=0.13 | p=0.29 |
| **24 h** | 48 | 0.05 | -0.08 | 48 | 0.20 | -0.02 |
|  |  | p=0.71 | p=0.60 |  | p=0.18 | p=0.88 |
| **48 h** | 48 | 0.08 | -0.01 | 46 | 0.19 | 0.00 |
|  |  | p=0.61 | p=0.93 |  | p=0.21 | p=0.98 |
| **72 h** | 45 | 0.13 | -0.02 | 45 | 0.22 | -0.02 |
|  |  | p=0.40 | p=0.92 |  | p=0.15 | p=0.98 |
|  |  |  |  |  |  |  |
|  |  | **NSE** |  |  | **T-tau** |  |
|  | n | Cg | Bg | n | Cg | Bg |
| **0 h** | 46 | -0.43 | -0.14 | 46 | -0.10 | -0.05 |
|  |  | **p=0.003** | p=0.38 |  | p=0.52 | p=0.75 |
| **24 h** | 48 | -0.07 | 0.00 | 48 | -0.01 | -0.07 |
|  |  | p=0.62 | p=0.97 |  | p=0.92 | p=0.62 |
| **48 h** | 46 | 0.09 | -0.03 | 46 | 0.07 | -0.01 |
|  |  | p=0.54 | p=0.84 |  | p=0.63 | p=0.97 |
| **72 h** | 45 | 0.18 | 0.10 | 45 | 0.12 | 0.04 |
|  |  | p=0.23 | p=0.53 |  | p=0.44 | p=0.78 |
